# Surface-water Interface Induces Conformational Changes Critical for Protein Adsorption: Implications for Monolayer Formation of EAS Hydrophobin

**DOI:** 10.3389/fmolb.2015.00064

**Published:** 2015-11-16

**Authors:** Kamron Ley, Andrew Christofferson, Matthew Penna, Dave Winkler, Shane Maclaughlin, Irene Yarovsky

**Affiliations:** ^1^Health Innovations Research Institute and School of Aerospace, Mechanical and Manufacturing Engineering, RMIT UniversityMelbourne, VIC, Australia; ^2^Australian Research Council (ARC) Research Hub for Australian Steel ManufacturingWollongong, NSW, Australia; ^3^CSIRO, Manufacturing FlagshipClayton, VIC, Australia; ^4^Institute of Pharmaceutical Science, Monash UniversityParkville, VIC, Australia; ^5^Institute for Molecular Science, Latrobe UniversityBundoora, VIC, Australia; ^6^BlueScope Steel Research LaboratoriesPort Kembla, NSW, Australia

**Keywords:** molecular dynamics, hydrophobin, anti-fouling, protein adsorption, silica surface, biofouling

## Abstract

The class I hydrophobin EAS is part of a family of small, amphiphilic fungal proteins best known for their ability to self-assemble into stable monolayers that modify the hydrophobicity of a surface to facilitate further microbial growth. These proteins have attracted increasing attention for industrial and biomedical applications, with the aim of designing surfaces that have the potential to maintain their clean state by resisting non-specific protein binding. To gain a better understanding of this process, we have employed all-atom molecular dynamics to study initial stages of the spontaneous adsorption of monomeric EAS hydrophobin on fully hydroxylated silica, a commonly used industrial and biomedical substrate. Particular interest has been paid to the Cys3-Cys4 loop, which has been shown to exhibit disruptive behavior in solution, and the Cys7-Cys8 loop, which is believed to be involved in the aggregation of EAS hydrophobin at interfaces. Specific and water mediated interactions with the surface were also analyzed. We have identified two possible binding motifs, one which allows unfolding of the Cys7-Cys8 loop due to the surfactant-like behavior of the Cys3-Cys4 loop, and another which has limited unfolding due to the Cys3-Cys4 loop remaining disordered in solution. We have also identified intermittent interactions with water which mediate the protein adsorption to the surface, as well as longer lasting interactions which control the diffusion of water around the adsorption site. These results have shown that EAS behaves in a similar way at the air-water and surface-water interfaces, and have also highlighted the need for hydrophilic ligand functionalization of the silica surface in order to prevent the adsorption of EAS hydrophobin.

## Introduction

Microbial adhesion plays a pivotal role in contamination and degradation in a variety of areas, ranging from biomedical (Campoccia et al., 2013; Desrousseaux et al., 2013; Harding and Reynolds, 2014) to surface coating (Díaz et al., 2007; Hasan et al., 2013) and marine (Flemming, 2011; Kamino, 2013) applications. Although the issues of biofouling and biofilms have been known for centuries, a fundamental understanding of the formation of these complex structures is still lacking, and presents one of the major issues in the modern world (Schmitt, 2009; Kamino, 2013; Nya, 2015), with costs to governments and industries of over a hundred billion dollars per year (Colautti et al., 2006; Schmitt, 2009; Schultz et al., 2011). Although the technologies to remove biofilms are improving considerably, there are significant limitations in reactive treatments due to the small length scales where biofilms are problematic. Examples of this are particularly evident in marine environments, where 25–50 μm biofilms on a ship hull increase hydrodynamic drag by 8–22% respectively (Townsin, 2003; Schultz et al., 2011), as well as health industries, where it is estimated that 20% of fatalities world-wide are due to infectious diseases, of which 80% are associated with biofilm formation (Prentice et al., 2004).

With these factors considered, it is not surprising that the focus of anti-fouling technologies has shifted to the design of surfaces that have the potential to maintain their clean state by resisting the non-specific binding of proteins and other foulants. However, significant limitations in these coating technologies arise due to our limited understanding of the interactions and behavior of microbes at interfaces. Experimental research has shown that the hydrophobicity of surfaces has significant effects on adhesion, with hydrophobic substrates generally incurring increased amounts of microbial adhesion in terms of increased numbers of attached cells, rates of attachment and binding strengths (Yiapanis et al., 2007; Krishnan et al., 2008; Chen et al., 2010). Conversely, hydrophilic surfaces that are highly hydrated have been shown to be more resistant to adhesion (Chen et al., 2010; Schwierz et al., 2012). This has been attributed to their ability to adsorb more water, which must be displaced before adhesion can occur (Mitik-Dineva et al., 2006; Bazaka et al., 2011). Nanostructured surfaces with alternating hydrophobic/hydrophilic characteristics have recently been shown to be able to either promote or inhibit protein adsorption (Hung et al., 2011), the phenomenon can potentially be exploited to design surfaces resistant to biofouling.

More recently there has been significant research into the behavior of interfacial water, and the critical role it plays in protein adhesion. At the surface-water interface, water has been seen to form two distinct “shells” which have significantly different properties to that of bulk water. The first shell is highly ordered and tightly bound, as water molecules form hydrogen bonds with the surface. A second layer is subsequently formed through hydrogen bonding with neighboring water molecules, resulting in a weakly ordered region. However, in areas with significant spacing between hydroxyl groups or high surface roughness, this interfacial layer often creates areas void of water, encouraging the adsorption of hydrophobic molecules (Notman and Walsh, 2009; Schneider and Ciacchi, 2012). It has been observed that the specific ordering of these shells play a pivotal role in the promotion or retardation of proteins and other contaminants adsorption. Specifically, as a protein approaches the surface, interfacial water layer undergoes structural changes which will help repel or promote adsorption of the protein (Argyris et al., 2011; Penna et al., 2014). These hydration forces have mainly been attributed to the surface heterogeneity, orientation, and local density of interfacial water (Kim and Cremer, 2001). This has inspired significant research into the behavior of interfacial water as a protein comes toward a surface, and strategies that can be adopted to prevent protein adsorption (Zheng et al., 2005; Leung et al., 2012; Hung et al., 2013; Yiapanis et al., 2014).

Hydrophobins are of particular concern in the field of biofouling. A low molecular weight (7000–9000 Dalton) family of proteins unique to filamentous fungi, hydrophobins have functionality in both producing a protective hydrophobic and waterproof layer around the fungi, as well as facilitating the adhesion of these species to surfaces (Linder et al., 2005). Hydrophobins are secreted by fungi in monomeric form, and possess the ability to spontaneously adsorb into stable amphipathic monolayers upon reaching an interface. These monolayers significantly alter the surface environment, reducing surface tension and altering the wettability of both hydrophobic and hydrophilic surfaces, conditioning them for further fungal adhesion including the production of hyphae (Wösten and de Vocht, 2000; Linder, 2009; Lo et al., 2014). There are two classes of hydrophobins based on the aggregates they form. Class I hydrophobins assemble into ordered rodlets with an amyloid-like structure that is incredibly robust, requiring strong acids to dissolve (Wösten, 2001). Class II hydrophobins are significantly less robust, dissolving in detergent and alcohol solutions, and lack rodlet morphology (Lugones et al., 1996; Paananen et al., 2003; Paslay et al., 2013). Because of their greater difficulty in removal, the focus of this study is on Class I hydrophobins, specifically, the Class I hydrophobin EAS, found in the fungus *Neurospora Crassa* (Kwan et al., 2006). EAS hydrophobin is comprised of a β-core region which comprises of three sets of anti-parallel β-sheets, with the overall structure maintained through four disulfide bonds. The protein is largely globular and highly disordered in bulk solution, however at the interface it is believed to undergo conformational rearrangement and form an intermediate state which is prone to aggregate into amyloid like monolayers (Macindoe et al., 2012).

To date there has been significant research on the behavior of EAS hydrophobin in bulk water solution and at the air-water interface which has shown several important properties, including the inability for EAS to aggregate in aqueous solution (Mackay et al., 2001; Macindoe et al., 2012) which has largely been attributed to the flexible, intrinsically disordered Cys3-Cys4 loop (De Simone et al., 2012). Although it has been previously shown that the Cys3-Cys4 loop is not required for monolayer formation (Kwan et al., 2008), at the air-water interface the Cys3-Cys4 loop was theoretically shown to stabilize into surfactant-like conformations, with hydrophobic residues being directed to the air, and hydrophilic residues to the water.

Despite the high interest and some significant research on hydrophobin at the air-water interface, both experimental and theoretical, to the best of our knowledge there have been no studies investigating the behavior of EAS hydrophobin with atomistic detail at the surface-water interface. Therefore, although some advances have shown significant value for anti-fouling technologies, many fundamental aspects of microbial adhesion have not yet been described. For example, certain microbes have a higher preference for hydrophilic surfaces rather than hydrophobic (Mittelman, 1996) while hydrophobins are able to adsorb strongly on surfaces regardless of hydrophobicity. To combat some of these deficiencies in understanding, molecular dynamics (MD) simulations and other modeling techniques have become increasingly popular (Dill and Maccallum, 2012; Karplus and Lavery, 2014). Thanks largely to advances in computational performance (Meredith, 2009) the value of all-atom and coarse-grained models in MD has significantly increased as researchers are now able to simulate experimentally relevant system sizes and timescales. This allows the investigation of proteins and peptides at surfaces, however newer issues arise with the limitations in parameters available that accurately describe the interactions of both organic and synthetic surfaces, and issues in adequate conformational sampling (Faver et al., 2012; Mobley, 2012) that restrict the simulations of relatively large protein-surface systems.

In this study we implement MD to get insights into the initial stages of monomeric adsorption of EAS hydrophobin on highly hydrated silica surfaces, in order to gain some understanding of the possible conformational changes that may be responsible for monolayer formation. Specific attention is paid to the behavior of both the Cys3-Cys4 and Cys7-Cys8 loops, due to their previously described behavior at the air-water interface (Kwan et al., 2006, 2008; De Simone et al., 2012). We also examine the behavior of water at the protein-silica interface, specifically the role of water bridged interactions that promote protein adhesion to the surface. These interfaces are prominent in both biomedical and industrial environments (Jimenez et al., 2012; Kobayashi et al., 2012; Puddu and Perry, 2014) and understanding the behavior and interactions in these systems at the nanoscale (Patel et al., 2012; Treuel and Nienhaus, 2012; Yiapanis et al., 2012) will be critical for the rational design of anti-fouling surfaces.

## Methodology

### Surface model

To allow for both experimental comparison and future surface modification we used a previously modeled silica surface (Yarovsky et al., 1995; Henry et al., 2006) originated from Garofalini et al. (Feuston and Garofalini, 1989). This represents a realistic, highly hydrated amorphous silica surface with a surface silanol density of 4.7 OH groups per nm^2^. The amorphous silica substrate displays a density of 2.6 g/cm^3^ (comparable to experiment), an average film thickness of 17 Å (in the *z* direction) and lateral dimensions of 81 Å (in both *x* and *y* directions). After adding a vacuum spacer in the *z* direction, the film was packed in a periodically replicated three-dimensional cell with periodic boundary conditions. During the subsequent simulations, the surface OH groups remained free to move, while the underlying SiO_2_ atoms were kept fixed at their initial *x, y*, and *z* coordinates. Systems were solvated as described below, and five replicas were simulated using MD for 20 ns in order to accumulate statistics for the surface water and bulk water behavior without the presence of a protein for comparison with the surface-protein interfacial systems.

### Solution protein model and force-field validation

The NMR solution structure of the class I hydrophobin EAS, determined by Kwan et al., was obtained from the PDB structure 2FMC (Kwan et al., 2006). The protein was protonated in zwitterionic form and simulated in a periodic box of 70 × 70 × 70 Å filled with explicit water and 2 counter-ions to maintain system neutrality. The system was simulated for 30 ns with five replicas using the CHARMM22 (Mackerell et al., 1998) force-field, and another five replicas using the CHARMM27 (Buck et al., 2006) force-field with CMAP corrections to refine the NMR structure as a benchmark for comparison between the solution and the surface-water interface behavior. In solution, the CHARMM22 protein models were seen to better maintain the β-core structure from the NMR data than the CHARMM27, as shown by the root mean square deviations (RMSD) of key areas (Supplementary Figure 1). For the β-core and Cys7-Cys8 loop, which are expected to be reasonably stable in solution, the CHARMM22 force-field simulations exhibited significantly lower RMSD than those for the CHARMM27 force-field. Conversely, for the highly mobile and flexible Cys3-Cys4 loop, the CHARMM22 force-field simulations exhibited a higher RMSD than CHARMM27.

On evaluation of secondary structure behavior over time (Supplementary Figure 2), it is noted that the CHARMM22 simulation leads to a slight diminishing in the anti-parallel β-sheets of the core region, however the Cys7-Cys8 structuring is maintained. The CHARMM27 force-field maintains the core region, however due to the unfolding of the Cys7-Cys8 loop in solution, the β-structuring in these regions was completely lost. For these reasons we have chosen to use the CHARMM22 force-field, as it gives a more experimentally consistent representation of the protein structure and behavior in solution.

### Surface-protein system simulations

**Figure 1:**
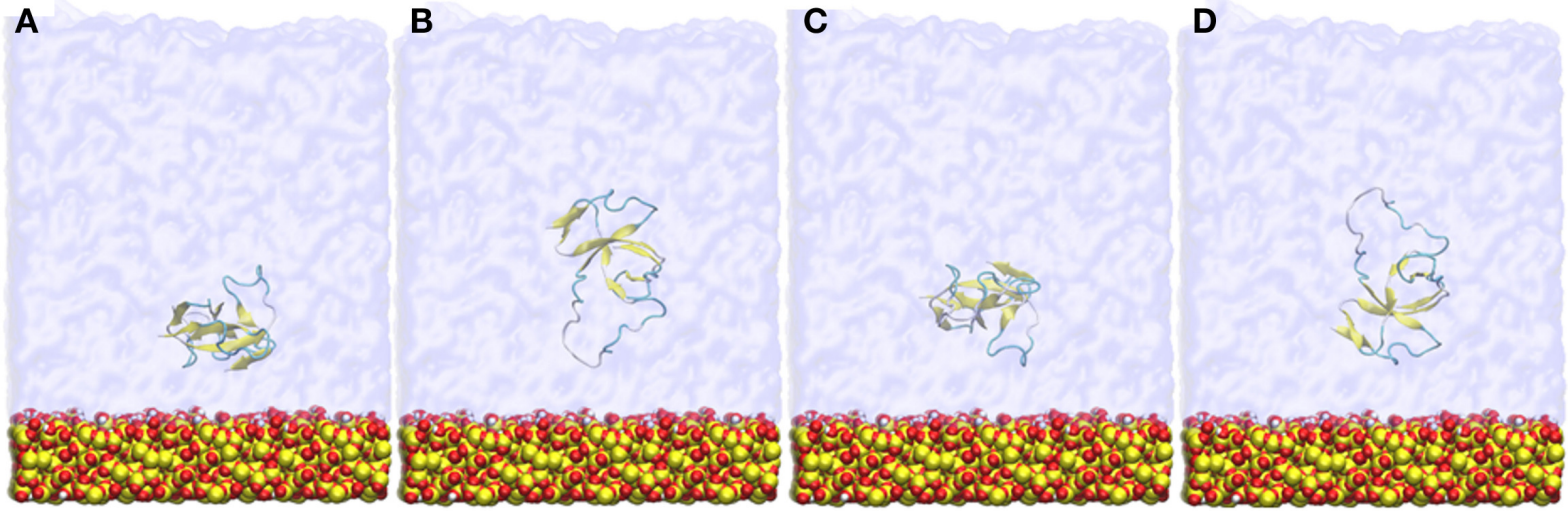
**Snapshots of the four different initial orientations (A–D) of EAS hydrophobin with respect to the silica surface**. The protein is positioned approximately 9 Å from the surface.

The protein initially equilibrated in bulk solvent as described above was placed approximately 9 Å from the surface, in four different initial orientations rotated 90° about the *y*-axis (as shown in Figure 1), to allow the investigation of binding orientations in spontaneous adsorption. The system was solvated using an explicit water layer of 80 Å thickness, with a 20 Å vacuum space above the water box added to create an air-water interface, and two counter-ions added to maintain system neutrality. Systems were first energy minimized using the conjugate gradient method. Following this, the water molecules were allowed to relax around the protein and surface by applying a short (2 ns) MD with the protein and surface constrained. Constraints were then removed and MD applied to the entire system for 50 ns to investigate the spontaneous initial adsorption events of the protein onto the silica surface. Simulations were repeated for each starting protein orientation with different initial velocities and equilibration was monitored by assessing the total energy trend. Whenever the protein adsorbed to the surface it happened spontaneously within the first 10 ns of the simulations. Data for analyses were collected from the equilibrated 20 ns of the simulation trajectory unless otherwise specified.

### Simulation settings

Simulations were performed using the LAMMPS (Plimpton, 1995) software with the CHARMM22 (Mackerell et al., 1998) force-field used for the protein, and the CHARMM-compatible Cruz-Chu (Cruz-Chu et al., 2006) silica parameters. The TIP3P (Jorgensen et al., 1983) water model was applied, with the SHAKE (Ryckaert et al., 1977) algorithm employed to constrain water bond length and angle. For the evaluation of non-bonded interactions, a twin-range cutoff of 0.8 and 1 nm were used for van der Waals interactions, with a 1 nm cutoff for electrostatics and the PPPM solver used to calculate the long-range damping effect. The energy minimizations were carried out using the conjugate gradient method with a convergence criteria of 1 × 10^−4^ energy tolerance and 1 × 10^−6^ force tolerance. MD was performed in the NVT ensemble using a timestep of 1 fs and a temperature of 298 K was maintained by a Nosé-Hoover thermostat (Nosé, 1984) with a 0.1 ps coupling time.

## Results

Five of the eight simulated systems adsorbed at the surface-water interface, whilst the other three adsorbed at the air-water interface. Our analyses will primarily focus on the systems that adsorbed at the surface-water interface, with particular emphasis on the behavior of the Cys3-Cys4 loop, Cys7-Cys8 loop and the role of interfacial water in the adsorption of EAS hydrophobin. This behavior will be compared to behavior in bulk and at the air-water interface to determine whether the physicochemical properties or water behavior are maintained, and validated against the existing and already detailed studies of EAS hydrophobin at the air-water interface (Kwan et al., 2008; De Simone et al., 2012).

### Protein binding at the surface-water interface

**Figure 2:**
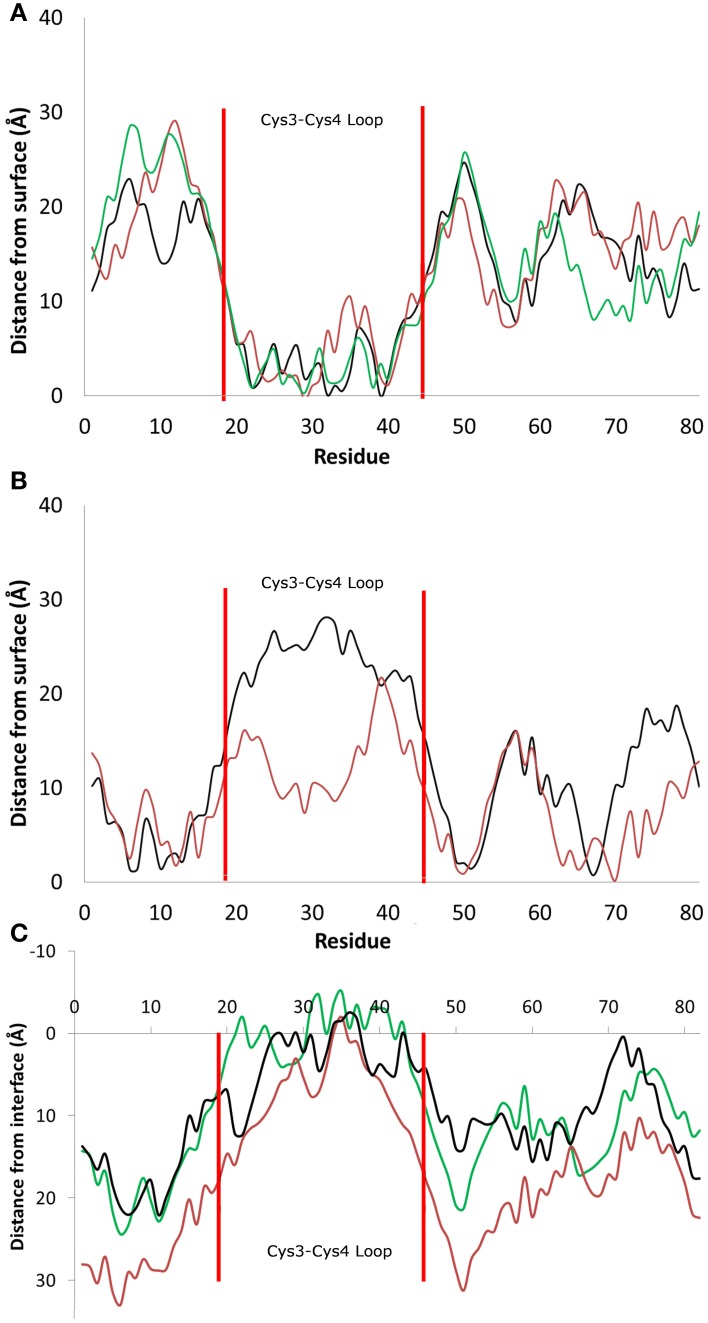
**Distance between the center of mass of residues and the average height of the surface hydroxyl groups for systems that adsorbed (A) through the Cys3-Cys4 loop (Residues 19 to 45, Binding Motif 1) and (B) with the Cys3-Cys4 loop in bulk water (Binding Motif 2). (C)** Distance between the center of mass and the average profile of the air-water interface. Different colors represent the initial protein orientation as shown in Figure 1. In Binding Motif 1, black and green colors were from orientation (A), red from orientation (D). In Binding Motif 2, red was from orientation (D) again, and black was from orientation (C).

Hydrophobin adsorption at the surface-water interface occurred spontaneously and we were able to identify two possible binding motifs at the interface, one in which adsorption occurs through the Cys3-Cys4 loop (Binding Motif 1, Figures 2A, **4B**), and another which has the Cys3-Cys4 loop away from the surface (Binding Motif 2, Figures 2B, **4C**). Interestingly, the initial protein orientation had minimal impact on the binding motif at the surface-water interface, as most systems experienced a slight reorientation in bulk water prior to adsorbing. The exception to this is the system that initially had the Cys3-Cys4 loop closest to the surface (Figure 1B), where the protein segregated to the air-water interface. This is most likely due to the Cys3-Cys4 loop initially contracting toward the β-core of the protein, resulting in increased distance between the protein and surface and therefore minimizing the attractive long-range interactions between them.

As expected, the interactions involved in both binding motifs are dominated by the surface-protein hydrogen bonding, due to the highly hydrophilic nature of the surface. There are, however, subtle differences in the specific nature of these hydrophilic interactions. In Binding Motif 1 (Figure 2A), the adsorption is largely due to the direct or water-mediated anchoring of residues 20–24 (QSMSG) and 38–40 (DLS), which are found at the beginning and end of the Cys3-Cys4 loop (residues 19–45). Within these groups, there are significant interactions between the surface hydroxyl groups and the hydrophilic side-chains of serine and aspartic acid, which encourage a tighter initial binding to the surface and subsequent interactions between the protein backbone and surface hydroxyls. Work by Sunde et al. (Kwan et al., 2008) has shown that removal of these residues inhibits surface activity and rodlet formation, however this only coincided with the mutated proteins EASΔ17 and EASΔ19 (EAS mutations with residues 24–40 and 23–41 removed respectively). It would be interesting to see if there was correlation between the mutation of residues 20–24 and 38–42 to glycine and a delay/inhibition of rodlet formation. In Binding Motif 2 (Figure 2B), persistent interactions with the surface occur in regions 6–7 (PN), 10–13 (SIDD), 50–52 (IGS), and 65–68 (VTNT). Unlike Binding Motif 1, these regions are dominated by backbone interactions, with very few side chain interactions having a significant occupancy over the simulation. Interestingly, this binding motif is almost identical to the binding of the EASΔ15 (EAS mutation with residues 25–39 removed) monomer at the air-water interface (Kwan et al., 2008). From our results it appears that the presence of the Cys3-Cys4 loop can inhibit the unlocking of the Cys7-Cys8 loop and thus monolayer formation, which will be discussed further below. It should be noted that due to the high flexibility, mobility and disordered behavior of the Cys3-Cys4 loop in bulk water, a broad distribution of distances from the surface can be seen in Binding Motif 2 (Figure 2B). This behavior has also been shown to occur at the air-water interface (Kwan et al., 2008).

### Specific interactions

**Figure 3:**
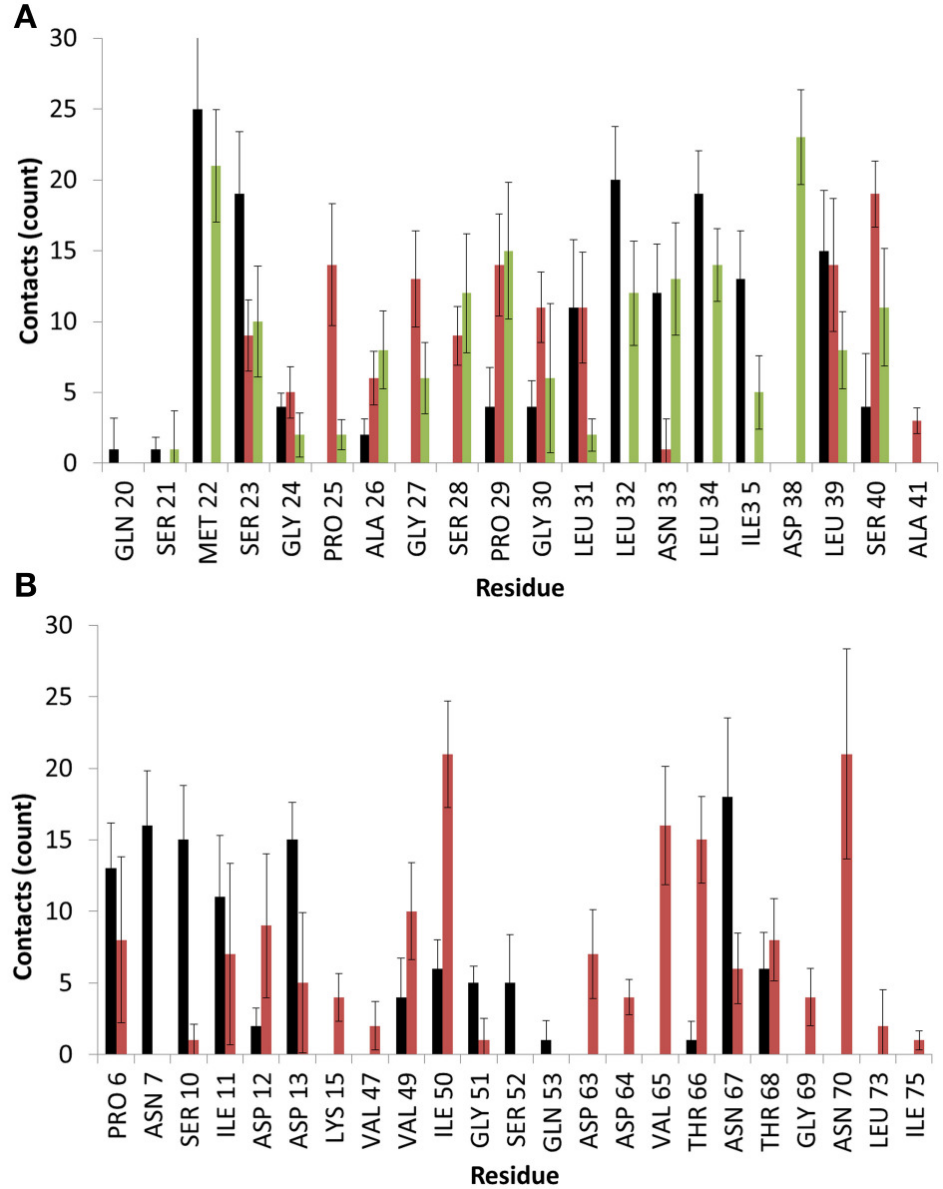
**Average number of contacts with the surface for EAS over the last 10 ns of simulation in binding motif (A) 1 and (B) 2**. Colors are matched to the residue-surface distance plots in Figure 2 and represent different simulation runs. Heavy atoms of a given residue are considered in contact with the surface if they fall within 4.5 Å of any surface atom.

To date there have been several studies by Walsh and colleagues on how the spacing of hydroxyl groups on silica surfaces effects the behavior of interfacial water, and how that influences the binding of hydrophobic and hydrophilic molecules and peptides (Notman and Walsh, 2009; Oren et al., 2010). Importantly, these works highlighted that larger spacing of hydroxyl groups on the surface would result in areas void of water. Free energy calculations have shown that it was energetically favorable for small hydrophobic moieties like methane to penetrate into these voids, where they would then be shielded by the surface interfacial water. This phenomenon was further explored on amorphous silica models with atomistic roughness, similar to those used in this study, by Schneider and Ciacchi (2012). In this study it was noted that these hydrophobic voids were present in larger volume due to surface cavities, which allowed penetration of hydrophobic side chains. On peptides which had alternating hydrophilic and hydrophobic residues, similar to those on EAS hydrophobin, it was noticed that adsorption was significantly enhanced as the hydropathicity of the interfacial water and voids could be matched, as well as allowing increased electrostatic interactions with the surface. In our simulations of EAS with the atomistically rough amorphous silica surface, we do indeed notice this phenomenon occurring. The average number of contacts for residues in contact with the surface during the last 20 ns of simulations for both binding motifs is presented in Figure 3.

As can be seen, significant contacts occur in hydrophobic residues such as Met22 in Binding Motif 1 and Ile50 in Binding Motif 2, as the hydrophobic sidechain penetrates the surface cavities. As with the aforementioned studies, these residues become shielded by surrounding interfacial water, which hold the residue sidechains in these voids, and allows further electrostatic interactions to occur as backbone atoms and shorter residues like glycine, proline and alanine come in contact with the surface hydroxyl groups, as well as charged and polar residues like serine, aspartic acid and asparagine which form direct interactions with the surface and surrounding water.

### Behavior of the Cys7-Cys8 and Cys3-Cys4 loops

**Figure 4:**
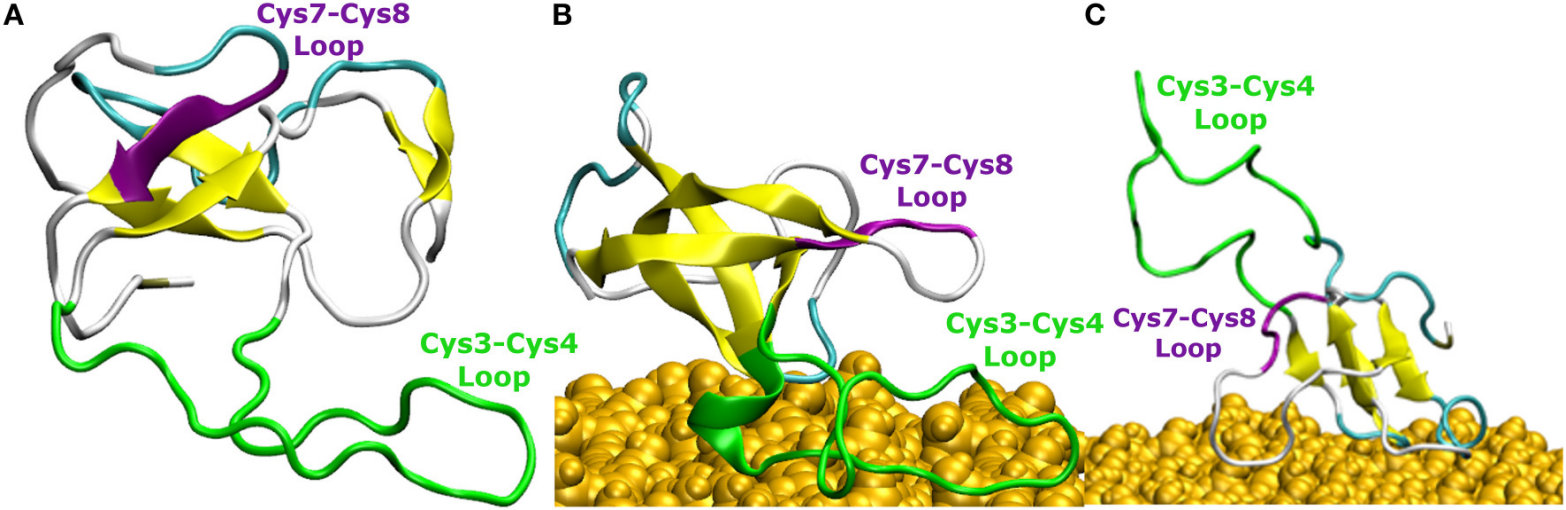
**Snapshots of EAS hydrophobin conformations (A) in bulk water, (B) at the surface-water interface when adsorbed through the Cys3-Cys4 loop (Binding Motif 1), and (C) at the surface-water interface with the Cys3-Cys4 loop in bulk (Binding Motif 2)**. Yellow arrows represent β-sheet structuring. The amyloidogenic region (F72-I75) is shown in purple and the Cys3-Cys4 loop shown in green.

The comparison of EAS hydrophobin features in bulk water and at the surface-water interface revealed several key differences. In bulk water, the amyloidogenic region (F72-I75) of the Cys7-Cys8 loop (Macindoe et al., 2012) interacts with the hydrophobic core of the protein, forming anti-parallel β-sheets in all five protein simulations in solution (Figure 4A). Interestingly, in two of the systems where the protein adsorbed through the Cys3-Cys4 loop to the surface-water interface (Binding Motif 1), we see significant interactions between adjacent strands in the Cys7-Cys8 loop, encouraging the formation of an exposed β-hairpin (Figure 4B). This intermediate state is consistent with the proposed model for EAS aggregation into monolayers at an interface (Macindoe et al., 2012).

**Figure 5:**
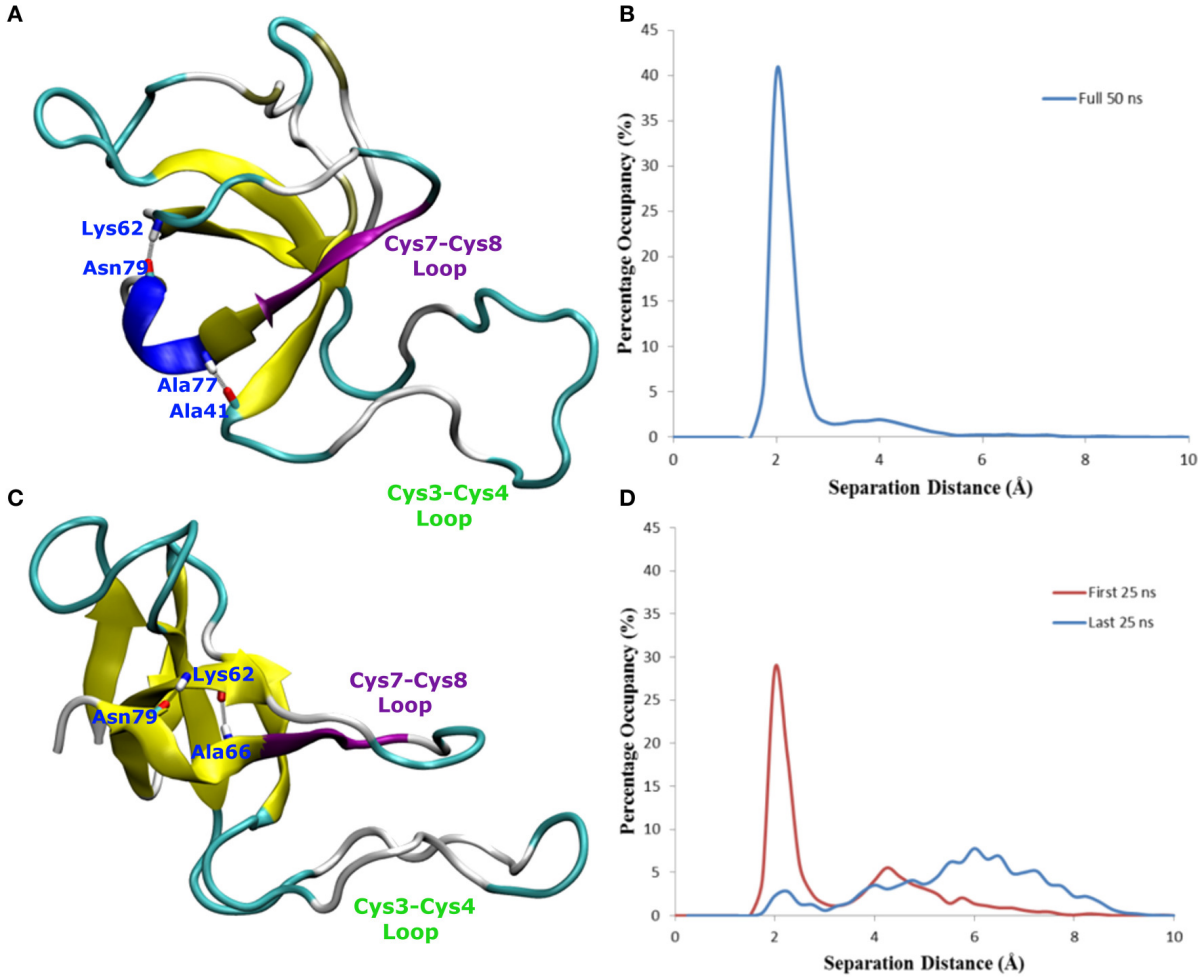
**Snapshots of EAS hydrophobin conformations highlighting hydrogen bonding between (A) residues Asn79 and Lys62, Ala77 and Ala41, resulting in a helical formation, (B) histogram showing the separation distance of residues Ala77 and Ala41 in system where Cys7-8 loop remains folded, and (C) residues Asn79 and Lys62, Asn76 and Lys62, encouraging the formation of the β-hairpin (D) histogram showing the separation distance of residues Ala77 and Ala41 in the system where Cys7-8 loop unfolds over time**.

In one of the systems that adsorbed through the Cys3-Cys4 loop (Binding Motif 1) a partial unlocking of the amyloidogenic region was observed, however interactions with the Cys3-Cys4 loop prevented the development of a β-hairpin structure. As can be seen in Figure 5, hydrogen bonding of residues near the C-terminus of EAS encourages the formation of either an alpha-helical structure (Figure 5A) which promotes the folded conformation of the Cys7-Cys8 loop seen in all bulk water simulations, or a β-sheet (Figure 5B), which encourages the unfolding of the Cys7-Cys8 loop. Upon conformational rearrangement at the surface-water interface, two of the three systems that adosrbed through the Cys3-Cys4 loop (Binding Motif 1) were able to overcome the energy barrier needed to break a critical hydrogen bond between residues Ala41 of the Cys3-Cys4 loop and Ala77 of the Cys7-Cys8 loop. Interestingly, there is a strong positive correlation between the degree of β-sheet formation for the Cys7-Cys8 loop and the number of contacts between the Ser40 (adjacent to the key Ala41 residue) sidechain and the surface (Figure 3A black, green, and red bars, and Supplementary Figures 3B–D, respectively). This interaction between Ser40 and the surface may be the first step in the process of unlocking the amyloidogenic region and subsequent hydrophobin monolayer formation. *In silico* mutation of the Ser40 to glycine could provide some insight into this relationship but is outside the scope of the current paper.

In systems which did not adsorb through the Cys3-Cys4 loop (Binding Motif 2) partial unfolding of the Cys7-Cys8 region was observed, however due to the aforementioned hydrogen bond (Ala41 to Ala77) persisting, there was no formation of a β-hairpin. This is likely to be due to the Cys3-Cys4 loop remaining in bulk solution, which enables it to retain the mobility and flexibility that is highly disruptive for the monolayer formation. As mentioned previously, this binding motif is consistent with the experimental observations by Sunde et al. (Kwan et al., 2008) of the binding of EASΔ15 at the air-water interface. Our finding also supports a more recent study by De Simone et al. (2012) which suggested that the primary role of the Cys3-Cys4 loop is to prevent the aggregation of hydrophobin in bulk water. Combined with the knowledge that these EASΔ15 proteins form rodlets that are almost indistinguishable from the native EAS (Kwan et al., 2008), we hypothesize that formation of an exposed β-hairpin is extremely likely in the event of the Cys3-Cys4 loop (A) being removed or (B) unfolding and interacting with the interface.

Changes in secondary structure on adsorption to the surface-water interface (Supplementary Figure 3) further show the disruptive influence the Cys3-Cys4 loop has on the protein conformation. In systems adsorbing through Binding Motif 2 (Supplementary Figure 3A), we see no structuring in the Cys7-Cys8 region due to disruptive interactions with the Cys3-Cys4 loop. This disruptive influence on the secondary structure is significantly reduced for systems interacting with the surface through Binding Motif 1. However, we also see how significant the Ala41-Ala77 interaction is. When this interaction is persistent (Supplementary Figure 3B) we see a stable 3_10_-helix in residues 76–79. Other than a temporary isolated β-sheet formation in residues 68–69 we see no significant changes in secondary structure. When this interaction is broken (Supplementary Figure 3C), we begin to see the significant enhancement in β-sheet formation, particularly in residues 73–81. On the system with no Ala41-Ala77 interaction (Supplementary Figure 3D) we see a very early and persistent β-hairpin formed.

### The role of structure and dynamics of interfacial water in hydrophobin adsorption

It has been well documented that highly hydroxylated silica surfaces, similar to those in this study, form significant hydrogen bonding with water molecules that enable them to retain an ordered interfacial water layer (Raschke, 2006). Furthermore, with studies showing the importance of water-mediated interactions for bio-fouling (Kim and Cremer, 2001; Zheng et al., 2005; Argyris et al., 2011; Leung et al., 2012; Hung et al., 2013; Penna et al., 2014; Yiapanis et al., 2014), we have investigated the specific involvement of water in the mechanisms of hydrophobin adsorption observed in our simulations. Studies have shown that proteins initially anchor to the surface-bound first hydration layer, resulting in significant restructuring of the interfacial water (Penna et al., 2014). It is then believed that intermittent interactions between these water molecules and protein residues encourage protein adsorption to the surface, which results in the displacement of these water molecules. Indeed, in our simulations, such dynamics have been observed as polar and charged amino acid residues experience transient interactions with the interfacial water molecules, encouraging rotation of the residues to maximize the contact surface area with the silica surface. As a result, due to the preferential interactions of the protein residues with the surface hydroxyl groups, the number of hydrogen bond sites available to water molecules is significantly reduced, leading to the observed displacement of water. In all three of the air-water simulations a partial unlocking of the Cys7-Cys8 loop occurred. However, this was perturbed by the formation of anti-parallel β-sheets between Ile75 of the Cys7-Cys8 loop and Leu43 from the Cys3-Cys4 loop, suggesting that although this intermediate transition is seen at both interfaces, the effects of multiple proteins at the interface must investigated to confirm whether this physicochemical transition is critical for the formation of monolayers.

**Figure 6:**
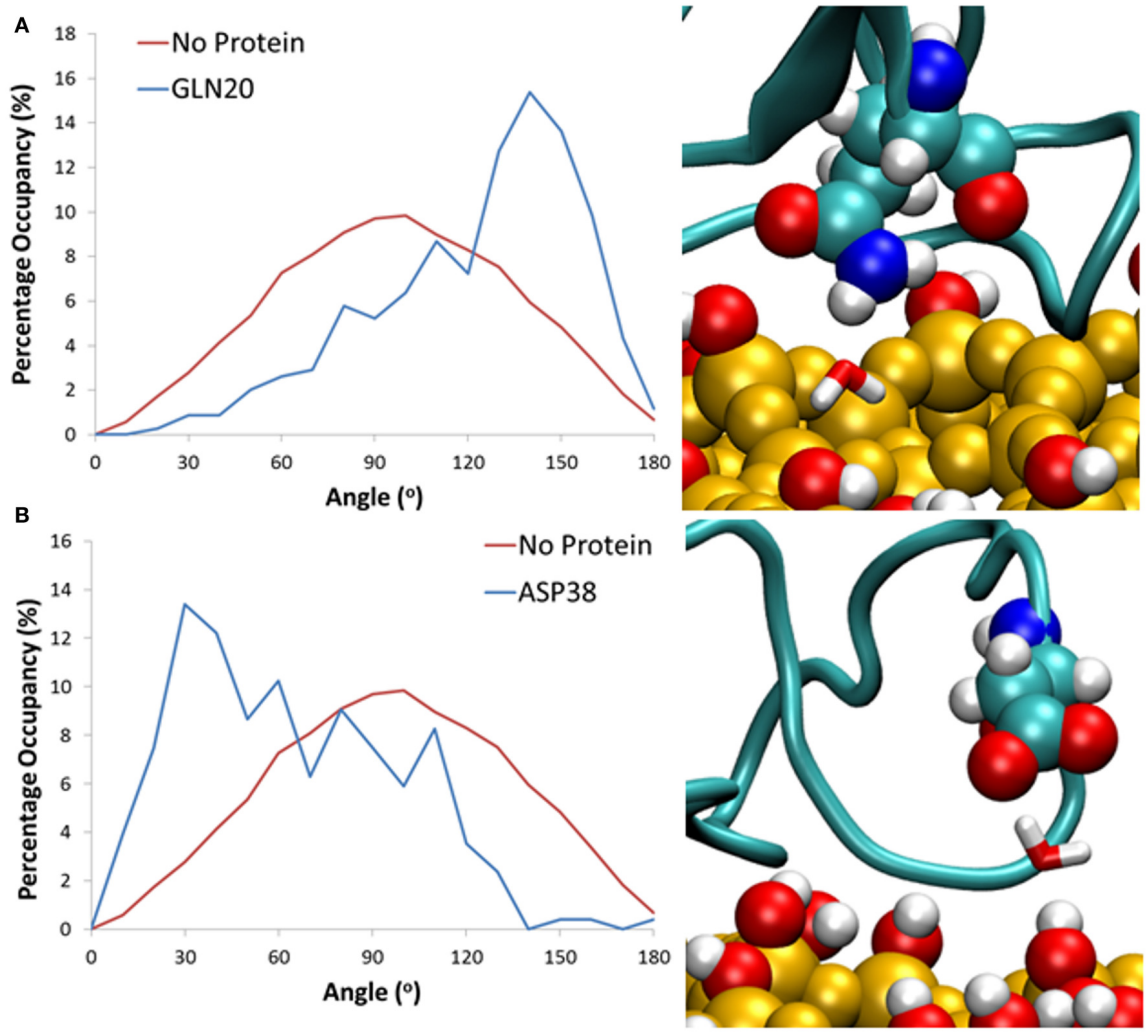
**Histograms showing the distribution of water dipoles around the surface with no protein (red) and with protein (blue) for water trapped between the surface and residues (A) Gln20 and (B) Asp38**.

In our simulations, the protein adsorption displaces water molecules as it adsorbs to the surface, resulting in surface areas significantly void of water, and a high level of occupancy around the protein. This displacement results in concentrated areas of high water occupancy around the protein adsorption site, as it is highly likely that the water molecules in this region are still forming “cushioned” interactions. Due to steric hindrances the sections of the protein around these areas of high occupancy cannot move closer to the surface, and instead form long lasting interactions with water, which results in slower water diffusion. To clarify this phenomenon, we have monitored the change in dipole moment orientation of water from interfacial regions around the surface, in a similar method to Hung et al. (2013). We have first computed the average water dipole moment at the water-surface interface without the influence of the protein. Water molecules within 3 Å of the surface hydroxyls have been considered, with an average distribution taken from the last 0.2 ns of simulation. As can be seen in Figure 6, the average dipole moment shows a broad symmetric distribution peaking at 90°. This distribution can be attributed to the high levels of surface hydroxylation, combined with a relatively smooth and rigid surface.

When comparing to systems with the protein at the surface-water interface, we consider cushioned water to be those that are within 3 Å of both the silica surface and a protein residue. All water molecules over the last 20 ns that fit this criterion have been considered, and the average dipole moment has been shown as a probability with an angle bin size of 10°. Results have shown that for these bridging interactions it is largely the side chain of charged and polar amino acid residues that have been involved in the formation of these long-lasting hydrogen bonds, enabling the water mediated protein-surface interactions. Specifically, for water trapped between polar residues such as Gln20 (Figure 6A) there is a significant shift in water dipole orientation toward 150°, which shows that the water molecule acts as hydrogen bond acceptor, resulting in its hydrogens pointing toward the surface. Conversely for negatively charged residues like Asp38 (Figure 6B) water acts as a hydrogen bond donor, resulting in a shift toward 30°, and hydrogen atoms pointing away from the surface. Residues that were more than 5 Å away from the surface are seen to have no effect on the dipole moment of cushioned waters. This is likely because the distance between the protein and surface does not allow for water molecules to form bridging bonds with both the protein and surface, and suggests that these water molecules still have a greater binding affinity to the protein rather than the surface.

**Figure 7:**
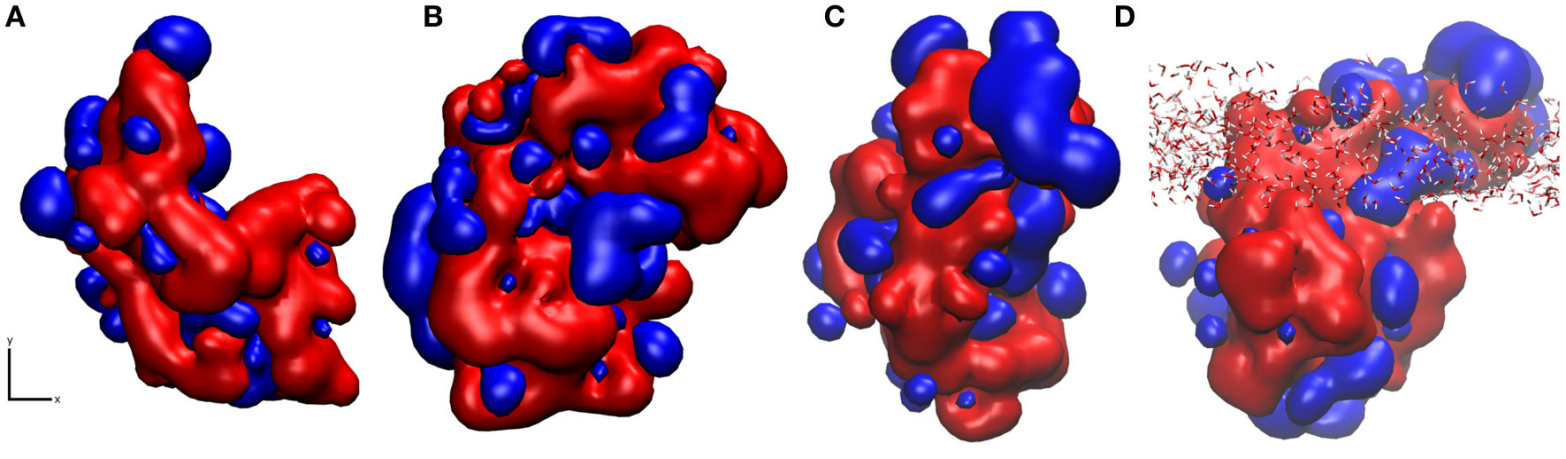
**Snapshots of hydropathicity for EAS showing the surface directly exposed to silica (x, y plane of the interface) in (A) Binding Motif 1 and (B) Binding Motif 2 and (C) at the air-water interface from top view and (D) rotated 90° around the x axis side view of (C) showing the interfacial water in all-atom detail**. Blue and red colors represent hydrophobic and hydrophilic regions respectively.

Using pyMLP (Broto et al., 1984; Laguerre et al., 1997), we have mapped the hydropathicity of the protein in both surface binding motifs (Figure 7) as well as at the air-water interface. As expected, the surface-water adsorption motifs (Figures 7A,B) are both dominated by hydrophilic interactions, with binding motif 2 (Figure 7B) showing slightly increased hydrophobic interactions due to the aforementioned surface cavitation effects. Conversely, at the air-water interface (Figures 7C,D), we see a more dominant hydrophobic surface, particularly outside of the air-water interface. Furthermore, adsorption results in significant water loss, particularly around hydrophobic residues (Table 1), where on average at least one water molecule per residue was lost on adsorption to the air-water interface, behavior not seen occurring at the surface-water interface. It is important to note that preferential bonding between silica and the charged groups of aspartic acid are likely to exclude water, and most likely the reason for the 5% difference. Also, for the large discrepancy in proline, there are only three proline residues in the studied area, and as seen in Supplementary Figure 4, one of which is in the middle of three hydrophobic side-chains, Leu34, Ile35, and Val37 extending out of the interface, and into the “air” environment, hence creating a significant loss in water.

**Table 1:** **Average loss of contacts with water for residues in the region Gln20-Ile50 for systems that adsorbed at the air-water and surface-water interface (Binding Motif 1), compared to the bulk environment, over the last 10 ns of simulation**.

**Amino acid type**	**Air-water (%)**	**Surface-water (%)**
Hydrophobic	34	17
Hydrophobic (large)	36	16
Polar	14	14
Proline	52	20
Aspartic acid	24	29

*A contact was defined as a water atom coming within 3 Å of the amino acid group types defined by Livingstone and Barton (1993). A detailed plot can be found in Supplementary Figure 4*.

**Figure 8:**
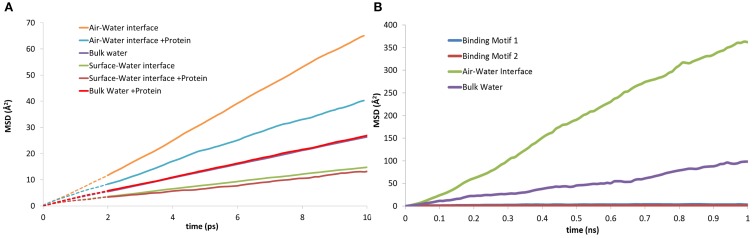
**Mean squared displacement (MSD) plots of: (A) water molecules at the, air-water, bulk water and surface-water interface; both with and without the presence of EAS hydrophobin. (B)** lateral MSD of the protein at the air-water interface, in bulk solution, and in both binding motifs at the surface-water interface.

The three-dimensional mean squared displacement (MSD) of water molecules at the surface-water interface and air-water interface with and without the presence of EAS have been compared to that of bulk water (Figure 8A). The curves are generated over a short-time domain (10 ps), with the gradient from a line of best fit plot proportional to the diffusion coefficient for the water molecules in the respective zones (Yiapanis et al., 2013). We observe a diffusion coefficient of 4.3 × 10^−5^ cm^2^/s for bulk water (Figure 8A), which is slightly higher than the reported 4.0 × 10^−5^ cm^2^/s diffusion coefficient for TIP3P with Ewald summation (Price and Brooks, 2004). As expected, the water at the surface-water interface (2.35 × 10^−5^ cm^2^/s) is significantly slower than bulk, due to stabilizing interactions with the silica surface. This is again reduced further when the protein is present (2.10 × 10^−5^ cm^2^/s), which shows that the protein does in fact trap water in the adsorption region, and limit the diffusion of water through aforementioned long polar and charged side-chain residues that are 5–6 Å from the surface, such as aspartic acid and serine. Conversely, at the air-water interface the diffusion is a factor of 10 faster without the protein, (1.15 × 10^−4^ cm^2^/s) slowing significantly in the presence of the protein (6.75 × 10^−5^ cm^2^/s). This behavior is in turn replicated for the mobility of the protein itself, where the MSD of the protein at the two interfaces can be seen in Figure 8B. At the surface-water interface the protein is practically immobile on the surface, with very little movement occurring once the protein is adsorbed. Comparatively at the air-water interface the protein moves at the interface freely, suggesting that there may be different mechanisms involved for hydrophobin monolayer formation at the air-water and surface-water interface, due to the vastly different surface hydropathicity and mobility of the protein. It may provide further support to the theory that the Cys3-Cys4 loop has surfactant-like behavior at the air-water interface (De Simone et al., 2012), especially considering the significant effects it has on the water diffusion coefficient.

These results demonstrate that the presence of water at the interface plays an important role in the mechanism of protein adsorption to the surface. Specifically, water mediates interactions between the surface hydroxyls and the protein by forming hydrogen bond bridges between the hydroxyls and the polar residues with medium to long side chains like serine, asparagine and glutamine, or the negatively charged residues of aspartic and glutamic acids. Furthermore, it appears that the surface silanol layer does not provide sufficient hydration retention thus enabling some water-mediated contacts as the main water layer is displaced and residual water molecules are trapped. This stabilizes the adsorption by secondary bridging interactions in addition to direct protein-surface interactions. We believe this deficiency could be overcome through surface functionalization by hydrophilic ligands that would be capable of maintaining a substantially thick and mobile hydration layer and prevent the protein from reaching the surface (Zheng et al., 2005; Chen et al., 2010; Leung et al., 2012; Yiapanis et al., 2014).

## Conclusions and perspectives

In this work we have shown two possible binding motifs for EAS hydrophobin at a hydrated silica surface during the early spontaneous adsorption events identified by MD simulations with atomic-level resolution. We found that for hydrophilic surfaces, the previously proposed aggregation state created by the unfolded Cys7-Cys8 loop is possible when hydrophobin adsorbs through residues 20–24 and 38–42 of the Cys3-Cys4 loop. It appears that there is a small energy barrier required to break a hydrogen bond formed between Ala41 and Ala77, which is necessary for the formation of the isolated β-sheet resulting from the unlocking of the Cys7-Cys8 loop. Furthermore, we have shown that the presence of areas void of water, due to roughness and hydroxyl spacing, allows the penetration of hydrophobic side chains, which bring the protein closer to the surface. Furthermore, there are significant interactions with the interfacial water layer which allow the formation of both intermittent and long-lasting interactions with this layer that seem to encourage rather than prevent the protein surface adhesion.

While this study does shed light on the monomeric hydrophobin behavior at the surface water interface, the conformational sampling enabled by the brute force MD is far from comprehensive yet it remains a challenge for systems of such sizes at all-atom detail. In addition, there is significant information lacking to provide specific strategies for the development of anti-fouling coatings. We believe that further investigation into the hydrophobin monolayer formation at various functionalized surfaces, including a combination of coarse-grained modeling to enhance the sampling and atomistic detail to understand the specific and non-specific interactions, is needed.

### Conflict of interest statement

The authors declare that the research was conducted in the absence of any commercial or financial relationships that could be construed as a potential conflict of interest.
