# Supplementary material for: Surface-water Interface Induces Conformational Changes Critical for Protein Adsorption: Implications for Monolayer Formation of EAS Hydrophobin
Source: Front Mol Biosci. 2015 Nov 16;2:64. doi: 10.3389/fmolb.2015.00064 (PMC4644811; doi:10.3389/fmolb.2015.00064)
Supplement: Supplementary file 1 [file DataSheet1.DOCX]

***Supplementary Material***

**Surface-water interface induces conformational changes critical for EAS hydrophobin monolayer formation**

Kamron Ley^1^, Andrew Christofferson^1^, Matthew Penna^1,6^, Dave Winkler^2,3,4^, Shane Maclaughlin^5,6^ and Irene Yarovsky^1,6^*

1 Health Innovations Research Institute and School of Aerospace, Mechanical and Manufacturing Engineering, RMIT University, Melbourne, VIC, Australia

2 CSIRO, Manufacturing Flagship, Clayton, VIC, Australia

3 Institute of Pharmaceutical Science, Monash University, Parkville, VIC, Australia

4 Institute for Molecular Science, Latrobe University, Bundoora, VIC, Australia

5 BlueScope Steel Research Laboratories, Port Kembla, NSW, Australia

6 ARC Research Hub for Australian Steel Manufacturing, Wollongong, NSW, Australia

Correspondence:

Irene Yarovsky
Health Innovations Research Institute
School of Aerospace, Mechanical and Manufacturing Engineering
RMIT University
GPO Box 2476
Melbourne, VIC, 3001, Australia

irene.yarovsky@rmit.edu.au

The RMSD plots (Supplementary Figure1) show that the CHARMM22 force-field presents as a much better representation of the solution structure than the CHARMM27 force-field. The rigid regions of the protein (Supplementary Figure1A) have a much lower RMSD using CHARMM22 than CHARMM27, showing a better representation of the NMR structure. Conversely, for the Cys3-Cys4 loop we see much higher RMSD in CHARMM22 than CHARMM27, however as this region is highly flexible and mobile, as well as intrinsically disordered, the CHARMM22 force-field is again more suitable.

When looking at the secondary structure reproduction for CHARMM22 (Supplementary Figure2B), we see slight diminishing in the anti-parallel β-sheets of the core region (residues 43-47, 52-54, 58-62, 79-82). However we do see the formation of an anti-parallel beta sheet in residues 73-76 as the Cys7-Cys8 loop remains folded to the β-core. For the CHARMM27 (Supplementary Figure2C) system we see much better retention of core and turn regions; however as the Cys7-Cys8 loop spontaneously unfolds in solution we see full loss of β-sheet secondary structure for this loop. We also see small segments occasionally forming a 3_10_-helix in residues 67-69.

Supplementary Figure 1. RMSD plots for NMR structures (blue), CHARMM22 (black) and CHARMM27 (red) of **(A)** protein and not Cys3-Cys4 loop; and **(B)** Cys3-Cys4 loop only. Systems are compared to the lowest energy NMR structure from Kwan et al. 2006. Dotted blue lines represent the standard deviation for the 20 NMR structures.

Supplementary Figure 2. Timeline graphs showing secondary structure of **(A)** 20 lowest energy configurations from NMR (PDB ID 2FMC, Kwan et al. 2006) **(B)** Secondary structure fluctuations for EAS in bulk solution using CHARMM22 **(C)** Secondary structure fluctuations for EAS in bulk solution using CHARMM27. Green color represents a turn structure, blue a 3_10_-helix, yellow a parallel/anti-parallel β-sheet and gold an isolated β-sheet.

Supplementary Figure 3. Timeline graph of secondary structure for Cys7-Cys8 loop regions of **(A)** Binding Motif 2 and the three different systems in Binding Motif 1 **(B-D).** For Binding Motif 1, **(B), (C), and (D)** correspond to the black, green, and red simulation runs in Figure 2 and Figure 3 of the main text. Green colors represents a turn structure, blue a 3_10_-helix, yellow a parallel/anti-parallel β-sheet and gold an isolated β-sheet.

Supplementary Figure 4. Average number of contacts with water for residues in the Cys3-Cys4 loop in bulk solution, and again at the air-water and surface-water interface over the last 10 ns of simulation. A contact was defined as a water atom coming within 3Å of the specified residue.
