# Supplementary figures and images for: Surface-water Interface Induces Conformational Changes Critical for Protein Adsorption: Implications for Monolayer Formation of EAS Hydrophobin
Source: Front Mol Biosci. 2015 Nov 16;2:64. doi: 10.3389/fmolb.2015.00064 (PMC4644811; doi:10.3389/fmolb.2015.00064)

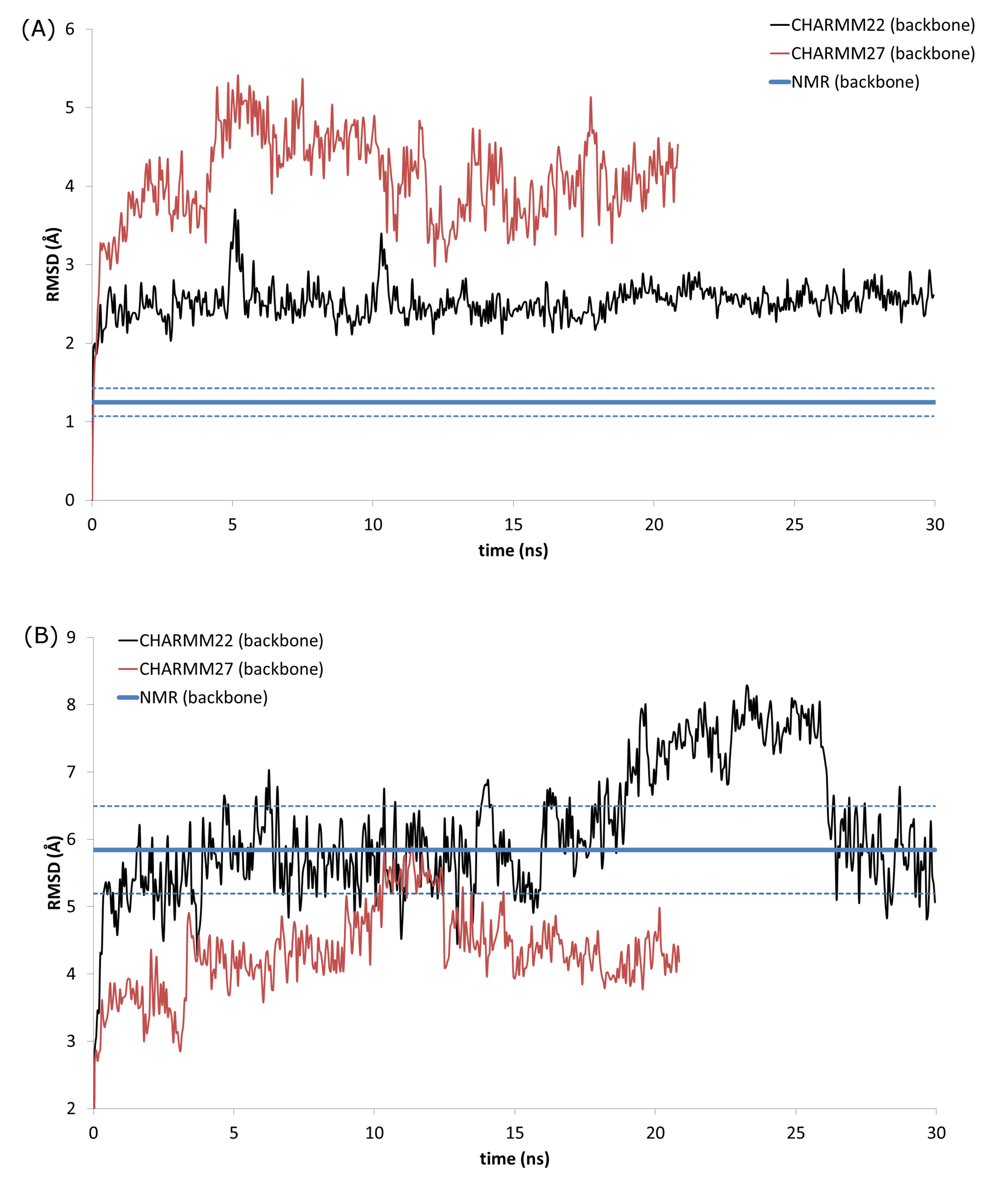

Supplement: Supplementary file 2 [file Image1.TIFF]

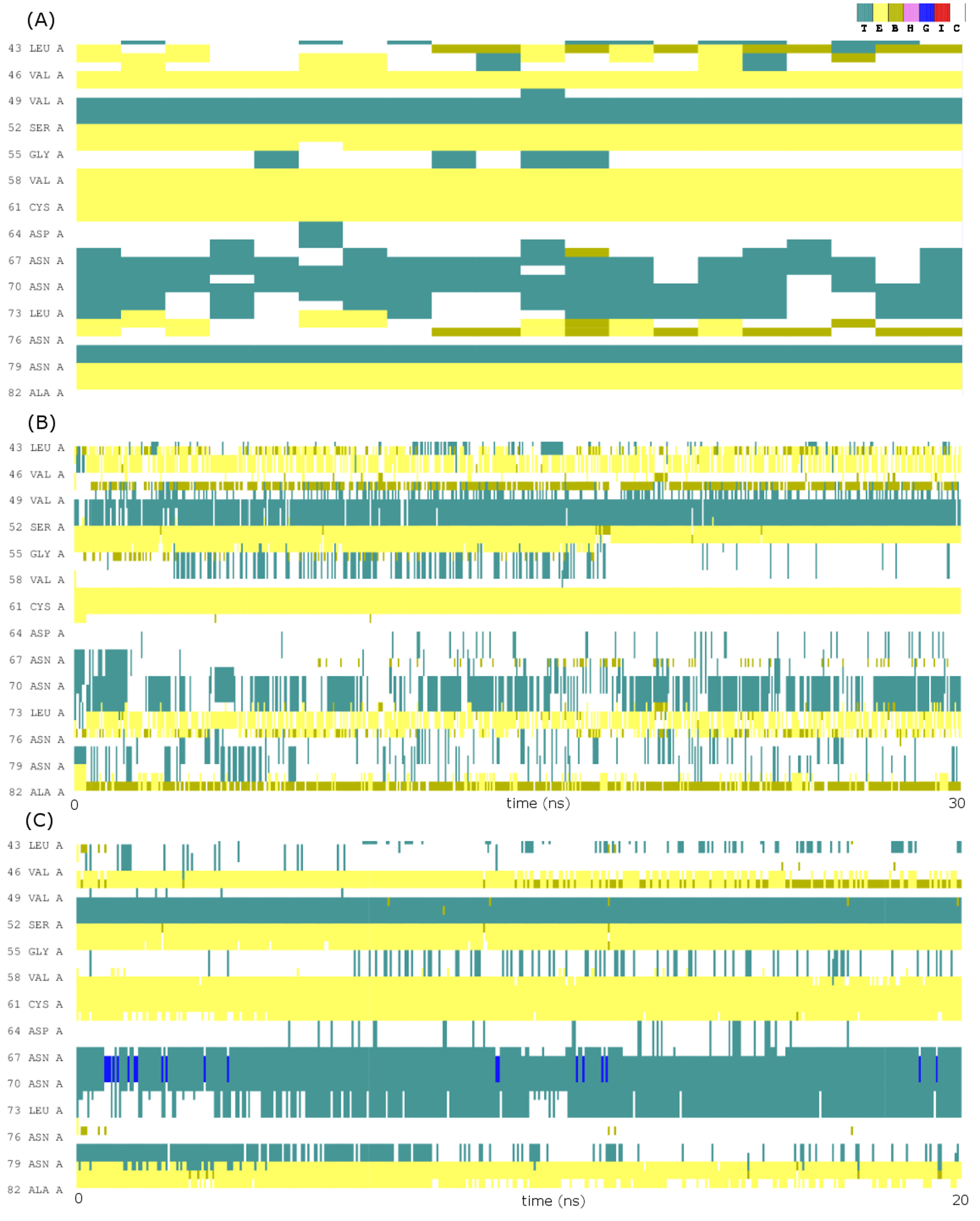

Supplement: Supplementary file 3 [file Image2.TIFF]

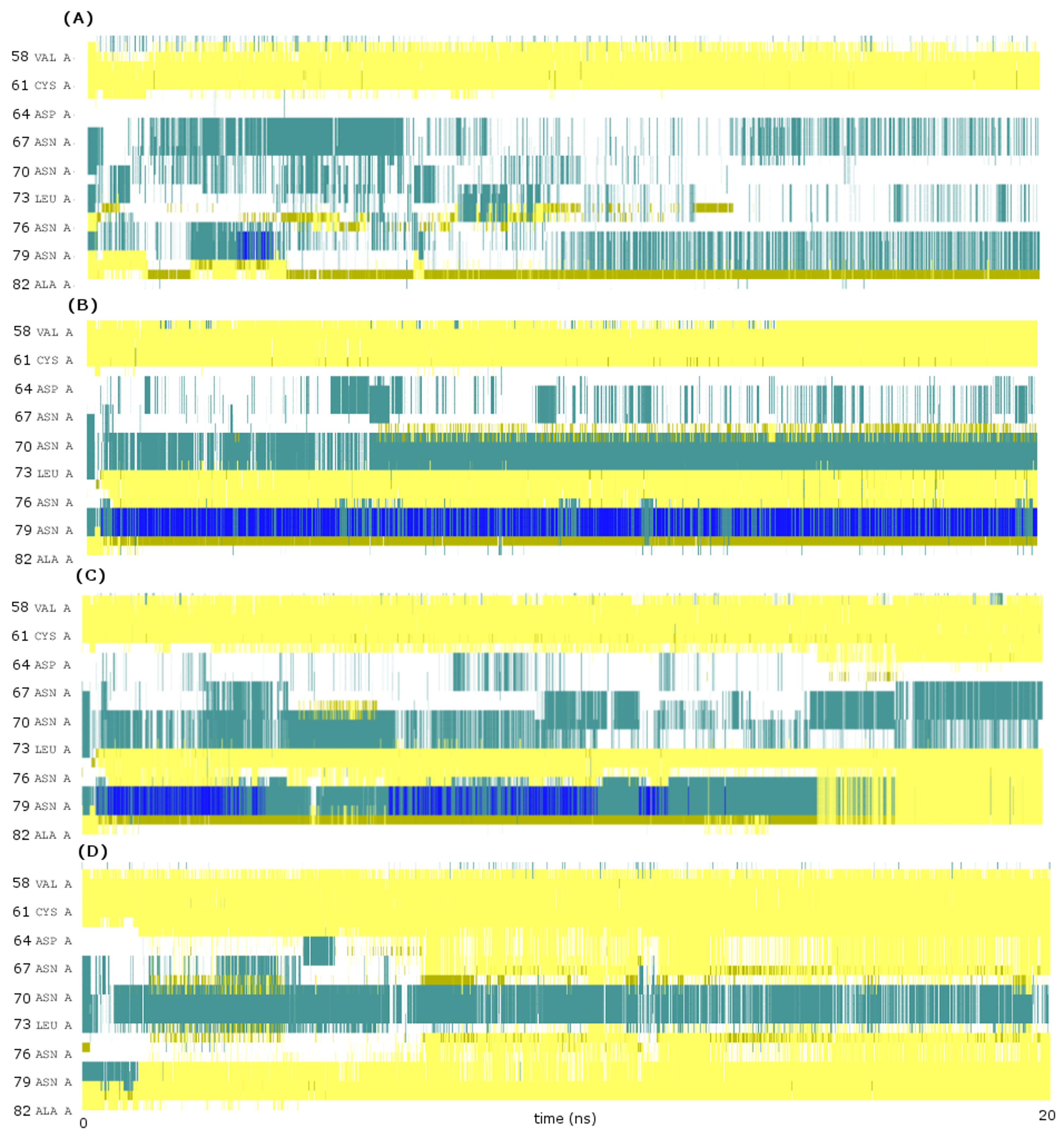

Supplement: Supplementary file 4 [file Image3.TIFF]

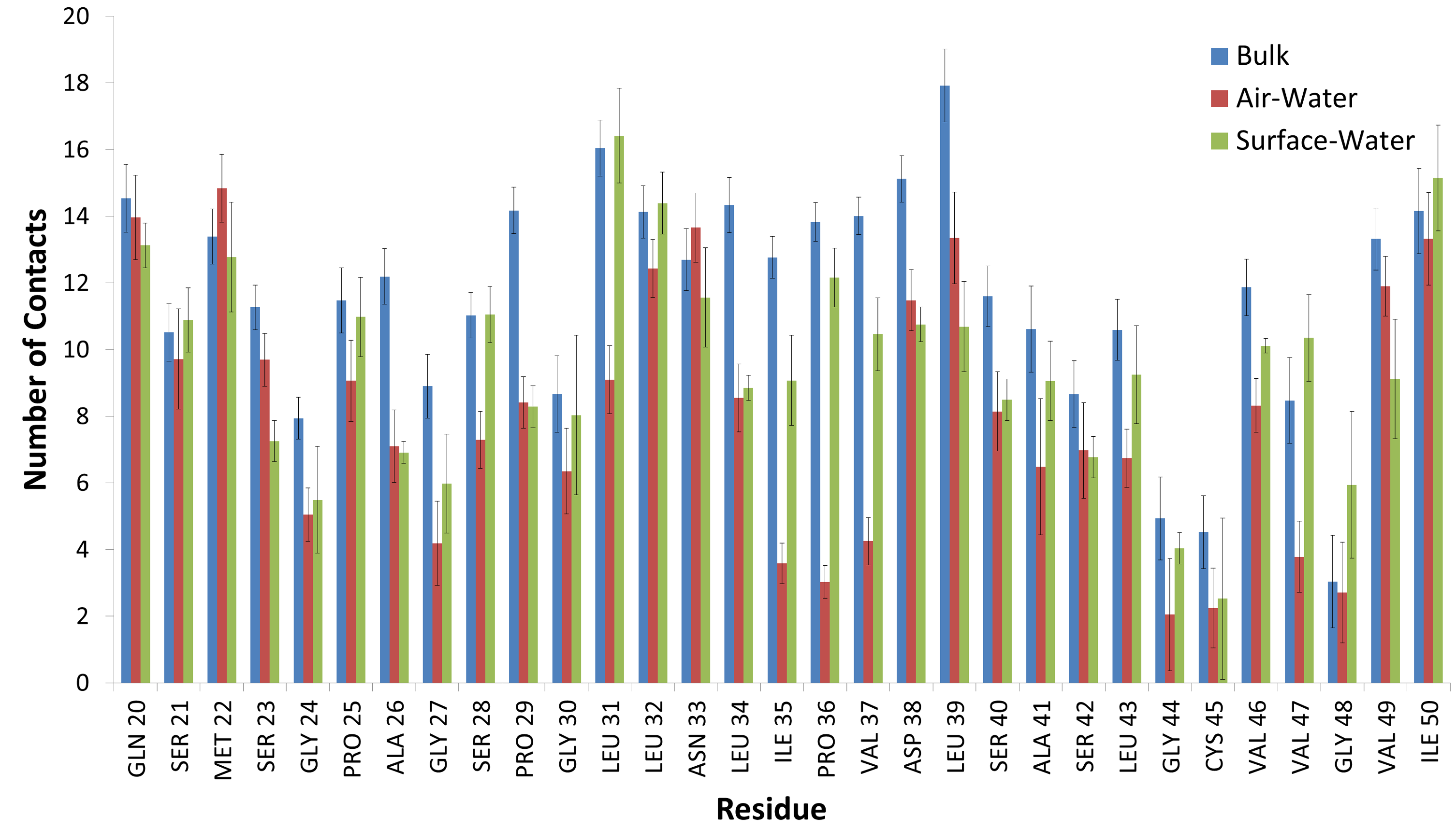

Supplement: Supplementary file 5 [file Image4.TIFF]
